# Identification of HCC-Related Genes Based on Differential Partial Correlation Network

**DOI:** 10.3389/fgene.2021.672117

**Published:** 2021-07-15

**Authors:** Yuyao Gao, Xiao Chang, Jie Xia, Shaoyan Sun, Zengchao Mu, Xiaoping Liu

**Affiliations:** ^1^Key Laboratory of Systems Biology, Hangzhou Institute for Advanced Study, University of Chinese Academy of Sciences, Hangzhou, China; ^2^Key Laboratory of Systems Health Science of Zhejiang Province, Hangzhou, China; ^3^School of Mathematics and Statistics, Shandong University, Weihai, China; ^4^Institute of Statistics and Applied Mathematics, Anhui University of Finance and Economics, Bengbu, China; ^5^Key Laboratory of Systems Biology, Center for Excellence in Molecular Cell Science, Institute of Biochemistry and Cell Biology, Shanghai Institutes for Biological Sciences, Chinese Academy of Sciences, Shanghai, China; ^6^School of Mathematics and Statistics, Ludong University, Yantai, China

**Keywords:** differential partial correlation network, hepatocellular carcinoma, functional module identification, multi-omics data, biomarkers

## Abstract

Hepatocellular carcinoma (HCC) is one of the most common causes of cancer-related death, but its pathogenesis is still unclear. As the disease is involved in multiple biological processes, systematic identification of disease genes and module biomarkers can provide a better understanding of disease mechanisms. In this study, we provided a network-based approach to integrate multi-omics data and discover disease-related genes. We applied our method to HCC data from The Cancer Genome Atlas (TCGA) database and obtained a functional module with 15 disease-related genes as network biomarkers. The results of classification and hierarchical clustering demonstrate that the identified functional module can effectively distinguish between the disease and the control group in both supervised and unsupervised methods. In brief, this computational method to identify potential functional disease modules could be useful to disease diagnosis and further mechanism study of complex diseases.

## Introduction

Hepatocellular carcinoma is the second most common cause of cancer-related death, with a low 5-year relative survival rates (Heimbach et al., 2018; Siegel et al., 2020) in the world. In recent years, a lot of research has been devoted to discovering disease mechanisms and disease-related genes for HCC. The traditional biological experiment takes a lot of time, cost, manpower, and material resources. To some extent, the methodology of computational biology may not be limited by these factors. Currently, many researchers study diseases by differential expression genes, considering genes with significant expression differences between cancer and normal tissue lead to cancer (de la Fuente, 2010). However, the onset of a complex disease is not caused by the expression change of a single gene but the dysfunction of the relevant system (Liu et al., 2016, 2019). Consequently, focusing only on differential expression of genes will lead to lots of key information of the disease being neglected. In comparison, the network-based approaches can discover disease progression by inspecting regulatory relationships between genes. Recently, a differential co-expression network was proposed to find out the alterations in network structure between normal and disease samples (de la Fuente, 2010). The protein interaction or gene regulation only occurred in one of the normal or disease status may be associated with the disease progress, which can be used to recognize disease-related changes in the regulatory system (Liu et al., 2012; Liu and Chang, 2016).

The conventional method of constructing a gene regulation network is usually to calculate the correlation coefficient between genes, but the Pearson correlation coefficient cannot be used to detect the direct regulation between genes (Zuo et al., 2014). The partial correlation coefficient can be used to eliminate the indirect regulation and keep the direct regulation between genes. In the practical calculation, the computational cost sharply increases when the order of correlation coefficients increases. When calculating first-order partial correlation, a fully connected network will take the most time, about O(*n*^3^), and it will compute faster in a sparse network (de la Fuente et al., 2004). Therefore, first-order partial correlation is appropriate to construct a gene regulation network even for large-scale data.

The disease progression is involved in biological processes on multiple layers, such as the genome, transcriptome, and epigenomics. For example, promoter hypermethylation can lead to the silencing of genes functioning in some cancer-related pathways, such as DNA repair and cell cycle regulation (Esteller, 2007). Information at different levels can complement each other. The joint analysis of multi-omics data can contribute to a better understanding of complex disease mechanisms and help the identification of disease biomarkers (Yan et al., 2018).

In this paper, we proposed a new method to identify disease genes by multi-omics data integration and network analysis. Based on gene expression data, we constructed a differential gene co-expression network. In particular, the gene co-expression network is not constructed by the Pearson correlation coefficient between genes but by the partial correlation coefficient, which reduces the indirectly related edges in a network. In addition, we also integrated DNA methylation data to identify edges that also change in methylation level. As supplementary information, single nucleotide variant data are used to prioritize genes according to the frequency of variation. Subsequently, a gene can be predicted as a disease-related gene if the gene occurred more variation and connect to more edges altered in both gene expression and DNA methylation levels. We applied the method to the HCC dataset from the TCGA database^1^. Finally, 15 genes are identified as disease-related genes, some of which have been already reported as tumor genes in the Cancer Gene Census^2^ (CGC) (Tate et al., 2019). Furthermore, the identified disease-related genes can distinguish tumor samples from normal samples by either classification or clustering. These results suggest that these predicted disease-associated genes can be used as effective modular biomarkers for HCC.

## Materials and Methods

### Data and Preprocessing

The RNA-Seq data, DNA methylation data, and SNP data were obtained from the TCGA database for HCC. The RNA-Seq data of HCC contains 371 tumor samples and 49 adjacent non-cancerous tissue samples as normal samples. The RNA-Seq data were normalized by the FPKM (The Fragments per Kilobase of transcript per Million mapped reads). We kept genes that were expressed in more than half of the total samples and can correspond to the Hugo Symbol for further study. For DNA methylation data, the Beta value was used to estimate the methylation level for each CpG site, and the sites that map to multiple genes or contain “NA” were filtered out. In addition, we used SNP data that are processed by MuSE Variant Aggregation and Masking workflow.

The protein-protein interaction (PPI) network of humans was obtained from the STRING database with version 11.0^3^ (Szklarczyk et al., 2019) which consisted of 11,759,454 interactions as background network. Each interaction in STRING PPI was assigned a confidence score ranged from 1 to 999 to reflect its reliability. We removed repeat interactions and kept the interactions with a confidence score greater than 500 from STRING PPI. Then, the interactions, which cannot be corresponded to the gene symbols in RNA-Seq data were removed from the PPI network. Finally, the background network with 582,168 interactions was obtained for further analysis.

### Construction of Differential Partial Correlation Network

We separated the RNA-Seq samples into normal and tumor groups and mapped the expression of genes into the background network. In each sample group, the partial correlation coefficient was calculated based on each edge in the background network, and two partial correlation networks were obtained in normal and tumor groups. We assumed that the two genes with a non-significant edge by partial correlation test do not interact or regulate in the corresponding group. Before calculating partial correlation, we first check whether there is a correlation between any two genes. The threshold of the *p*-value for the Pearson correlation coefficient was set adjusted value 0.01 using the Benjamini and Hochberg procedure method. It means that the edges with the adjusted *p*-values less than 0.01 were reserved, and the edges with *p*-values greater than or equal to 0.01 were ignored in the new network (Pearson correlation coefficient network, PCCN). In order to exclude the interference of indirect edges in PCCN, the partial correlation coefficient was computed for each edge in PCCN.

The partial correlation coefficient can test whether the correlation between two variables is linked to the third controlled variable. It is beneficial to remove the influence of the third controlled variable and only obtain the direct correlation between the two variables. For each reserved edge, the partial correlation coefficient can be calculated:

(1) $$r_{i\,j\,\left(k\right)}=\frac{r_{i\,j}-r_{i\,k}\,r_{j\,k}}{\sqrt{1-r_{i\,k}^{2}}\,\sqrt{1-r_{j\,k}^{2}}}$$

Where *i*,*j*,*k* are three genes on the PCCN, *r*_*ij*_ is the Pearson correlation coefficient between gene *i* and gene *j*, *r*_*ik*_ is the Pearson correlation coefficient between gene *i* and gene *k*, *r*_*jk*_ is the Pearson correlation coefficient between gene *j* and gene *k*, and *r*_*ij(k)*_ represents the partial correlation coefficient of gene *i* and gene *j* controlled by gene *k*.

The statistic *t* is computed with the method proposed by Weatherburn (1968):

(2) t=ri⁢j⁢(k)⁢N-q-21-r2i⁢j⁢(k)

**FIGURE 1:**
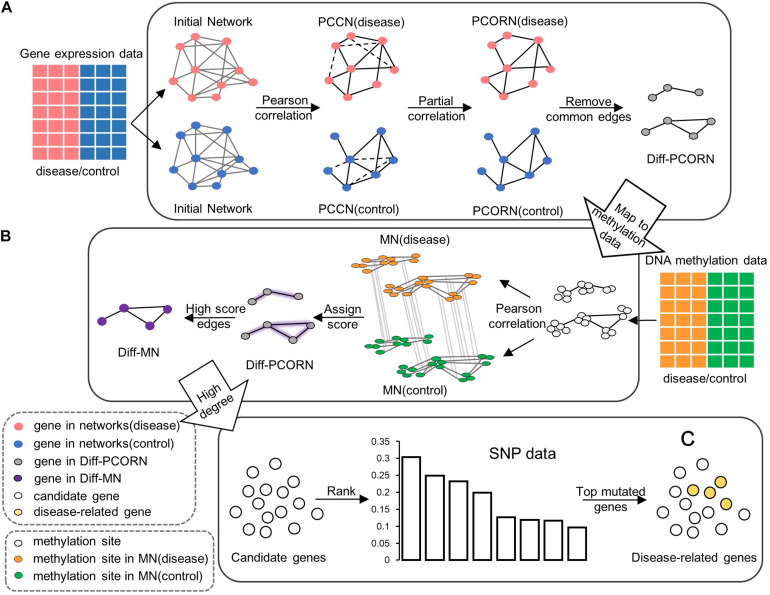
The flowchart for data integration. **(A)** Construction of differential partial correlation network (Diff-PCORN). After constructing the initial network based on the PPI network, the edges with a significant Pearson correlation are kept (adjusted *p* < 0.01) as the PCCN. Then, PCORN is constructed by deleting the non-significant edges (dotted line in PCCN) with adjusted *p*-values of partial correlation greater than or equal to 0.05. Remove edges in both PCORN (disease) and PCORN (control). **(B)** Construction of differential methylation network (Diff-MN). Pearson correlation coefficient can be calculated between methylation sites if there are interactions between their corresponding genes. Every edge is assigned a score by the maximum value of correlation difference between MN (disease) and MN (control). The edge with a score greater than 0.7 forms the Diff-MN. **(C)** Rank the candidate genes according to the variation frequency and the top 15 genes are identified as potential disease-related genes or module biomarkers.

Where *N* is the sample size and *q* is the order of partial correlation coefficient. Then, we calculated *p*-values by Student’s *t*-test and adjusted *p*-values by the Bonferroni method. The edges with the adjusted *p*-values < 0.05 were retained and the partial correlation networks (PCORN) were constructed by collecting the significant edges in normal and tumor status. The differential edges between PCORN in normal and tumor groups can reflect disease-specific alterations between normal and tumor status. So, the differential partial correlation network (Figure 1A) between normal and tumor status was constructed for detecting tumor-related genes, and we called it Diff-PCORN.

### Construction of Differential Methylation Network

In the same way, we also divided methylation data into normal and tumor groups. Each methylation site was mapped into a gene, and each gene may include more than one methylation site. The methylation network (MN) can be constructed by calculating the Pearson correlation coefficient between methylation sites, which correspond to different genes in Diff-PCORN. Two methylation networks were, respectively, constructed using the methylation data in normal and tumor groups. Then, for any pair of two methylation sites located in two genes of an edge, their correlation coefficient was compared between methylation networks in normal and tumor status, and the difference was regarded as the differential methylation score for this edge. If the differential methylation score of an edge is greater than 0.7, the edge should be reserved to compose a differential methylation network which was named Diff-MN (Figure 1B). When one gene was mapped to multiple methylation sites, more than one differential methylation score may be computed for an edge. In that situation, the maximum was retained as the differential methylation score.

### Data Integration and Disease-Related Genes Identification

In SNP data, genes were ordered by their variation frequency. For each sample, if mutations occurred on one or more sites in a gene, the gene was considered mutated in this sample. For each gene, its variation frequency was defined by the ratio of samples in which it has mutated to all samples in SNP data.

A three-step process was used to identify the potential disease-related genes for HCC. Firstly, the genes with a degree greater than 30 in the Diff-PCORN were chosen as the first candidate gene set. Secondly, the genes with a degree greater than 15 from the Diff-MN were chosen as the second candidate gene set. Thirdly, we obtained the overlapped genes between the two candidate gene sets and ranked the overlapped genes using ascending variation frequency (Figure 1C). The top 15 genes with the most frequent variation were identified as potential disease-related genes or module biomarkers (if variation frequency is the same in two genes, the gene with a greater degree in Diff-PCORN was chosen first).

### Validation of the Identified Disease-Related Genes

In order to validate the ability of disease-related genes to recognize cancer samples, we used them to distinguish normal samples from tumor samples. Support vector machine (SVM) algorithm was utilized for sample classification with the expression of the disease-related genes. In addition, the receiver operating characteristic (ROC) curve and the area under the ROC curve (AUC) were used to evaluate the performance of classification. Furthermore, to test whether the disease-related genes could identify samples in unsupervised learning, we utilized the hierarchical clustering method to distinguish normal and tumor samples. The single linkage with cityblock distance (Ren et al., 1998) was used in the clustering method, and the clustering result was visualized by heat map. SVM and ROC curve were implemented by Scikit-learn package (Python machine-learning library) (Pedregosa et al., 2011). Hierarchical clustering was implemented with the SciPy package^4^. Meanwhile, another independent liver cancer dataset from the GEO database (ID: GSE14520) was used to validate the availability of the potential disease-related genes (Roessler et al., 2010, 2012; Zhao et al., 2015; Sun et al., 2017; Wang Y. et al., 2019).

### Functional Verification of Module Biomarkers

The hypergeometric test was utilized to estimate the enriched significance of the module biomarkers to known tumor genes from the CGC database. The formula of the hypergeometric test is as follows:

(3) P(X≥x)=1-∑k=0x-1(Mk)⁢(N-Mn-k)(Nn)

Where *N* is the total gene number of RNA-Seq dataset, *M* is the number of known cancer genes, *n* is the number of the potential disease-related genes that we identified, *x* is the number of genes that overlap between known cancer genes and identified potential disease-related genes, (Mk) is a combinatorial number that represents all the combinations about selecting *k* elements out of *M* elements without repeating, and *P* is the statistical significance of the enrichment test. The enrichment analysis was also tested for the potential disease-related genes in hepatocellular carcinoma and cancer pathway from the KEGG database.

## Results

### Identifying Disease Genes Across Multiple Differential Networks

The disease-associated genes identified from gene expression data, DNA methylation data, and SNP data in disease may cause changes in these three aspects coordinately. Thus, we built the network gradually and integrated the three datasets to find out the potential cancer genes.

There are 582,168 edges and 16,264 nodes on the PPI network from the STRING database after filtering the RNA-Seq data. We separately calculated the Pearson correlation coefficient based on the background networks in the tumor group and normal group and reserved the edges with a significant correlation coefficient (adjusted *p* < 0.01). In the tumor group, 332,092 edges and 15,626 nodes were retained; similarly, 206,361 edges and 14,114 nodes were kept in the normal group. Although the retained edges are significant, the direct correlation and indirect correlation are still indistinguishable. In order to filter out the indirect edges from the network, we utilized partial correlation analysis and eliminated the non-significant edges with the adjusted *p*-values of partial correlation coefficient greater than or equal to 0.05. In this way, we obtained PCORNs with 58,195 edges and 15,353 nodes in the tumor group and 20,290 edges and 12,841 nodes in the normal group. After removing 10,646 common edges in both tumor and normal status, Diff-PCORN consisted of the remaining edges only in one PCORN in either tumor or normal. There were 15,439 nodes and 57,193 edges in Diff-PCORN, of which 47,549 edges came from the tumor network, and another came from normal. We considered that the more edges a gene connected in Diff-PCORN, the more likely it played an important role in tumor progression. Therefore, 269 genes with a degree greater than 30 were selected from Diff-PCORN as a candidate tumor-related gene set. Then, we performed functional enrichment analysis for these genes using DAVID (Huang et al., 2009), and they are enriched into the cell cycle, adherens junction, and viral carcinogenesis pathways (Supplementary Table 1).

In addition, some edges were also altered from the normal to tumor group in the methylation space. In total, 44,034 edges with 13,052 nodes of Diff-PCORN corresponded to the methylation data. Since one gene may be mapped to more than one methylation site, we used the most significant change of the methylation site pair to represent the score of an edge. For example, if A-B is an edge in Diff-PCORN, gene A includes two methylation sites a_1_ and a_2_, and gene B includes two methylation sites b_1_ and b_2_. Consider four relationships between paired methylation sites: (a_1_, b_1_), (a_1_, b_2_), (a_2_, b_1_), and (a_2_, b_2_). For the methylation site pair (a_1_, b_1_), we calculated the correlation coefficient both in tumor group (*r*_*tumor*_) and normal group (*r*_*normal*_), respectively, and then, the absolute value of the difference between those two groups (|*r*_*tumor*_−*r*_*normal*_|) was calculated as a correlation difference of (a_1_, b_1_). The correlation difference of (a_1_, b_2_), (a_2_, b_1_), and (a_2_, b_2_) can be computed in the same way. Next, the maximum correlation difference is assigned as the differential methylation score of edge A-B in Diff-PCORN. Diff-MN was composed of edges with scores of more than 0.7, hence 20,570 edges with 9,978 nodes were kept in Diff-MN. The genes with a degree greater than 15 in Diff-MN were chosen as the second candidate gene set which contained 244 potential disease genes and they are enriched into pathways of adherens junction, proteoglycans in cancer, and pathways in cancer (Supplementary Table 2).

Applying the above criteria, there are 269 genes from the first candidate gene set and 244 genes from the second candidate gene set. And, 141 overlapped genes were obtained from the two candidate gene sets. According to the frequency of variation in SNP data, the top 15 genes from the overlapped genes were identified as the final disease-related genes or module biomarkers (Supplementary Table 3). For gene functions, we found that some of the disease-related genes are associated with HCC. For example, *BPTF* promotes the growth of cancer cells by regulating the expression of human telomerase reverse transcriptase, and its high expression is associated with advanced malignancy (Zhao et al., 2019). *DHX9* encodes an RNA helicase, which is an essential factor in the regulation of Hepatitis B virus DNA replication, virus circular RNA, and virus protein levels (Sekiba et al., 2018; Shen et al., 2020). In addition, when the interaction of *DHX9* with *CDK6* is prevented by a specific lncRNA, the growth of HCC will be promoted (Wang Y. L. et al., 2019). Furthermore, after enrichment with DAVID, module biomarkers are mainly gathered in some pathways, such as HTLV-I infection, cell cycle, hepatitis B, viral carcinogenesis, and microRNAs in cancer pathway, which implies that HCC may be linked to viral factors (Supplementary Table 4).

**FIGURE 2:**
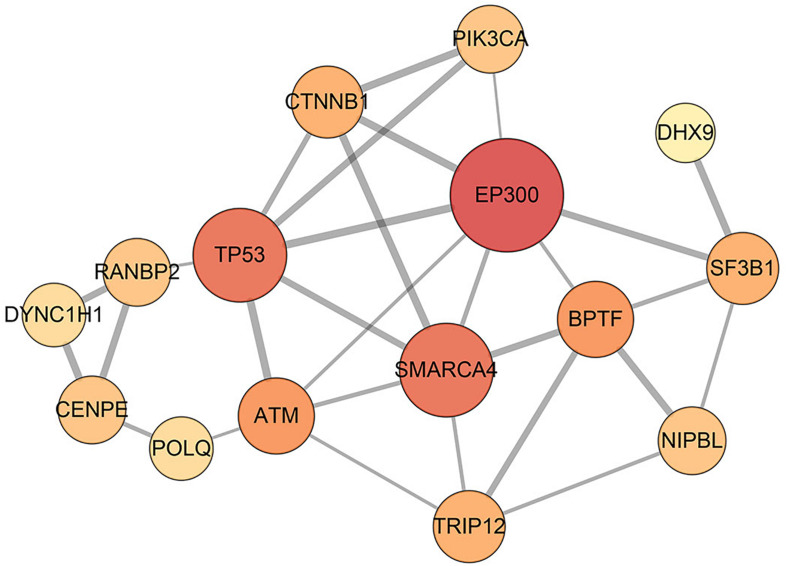
Interaction network of disease-related genes. In total, 29 interactions among 15 disease-related genes are identified by STRING database, and they can be considered as a disease module. The edge with a higher combined score from the STRING database is wider, and the node with the higher degree is bigger.

Furthermore, the predicted module biomarkers connected to each other and their interactions in the STRING database (confidence > 0.5) are shown in Figure 2. In different conditions, disease-related genes and their corresponding interaction partners demonstrate different structural compositions (Supplementary Figures 1–4). In PCORN (tumor), disease-related genes and their partners constituted a subnetwork with 833 edges and 715 nodes. Meanwhile, another subnetwork of PCORN (normal) was constructed by 109 edges and 122 nodes. We performed pathway enrichment analysis using disease-related genes and their interaction partners. The results show all the pathways in the normal group can be found in the tumor group; however, some pathways, such as the cell cycle, viral carcinogenesis, and p53 signaling pathway, are only enriched in the tumor group (Supplementary Tables 5, 6). In addition, the connection of module biomarkers is different in the normal and tumor groups. For the partial correlation network, we can see from Supplementary Figure 1 that there is no edge between disease-related genes in the normal group; however, 9 out of 29 edges from identified disease module still exist in the tumor group. And we considered that these edges may be associated with tumors. Although the mechanisms of these interactions are unclear at present, some genes of these 9 edges have been reported that are involved in the HCC process, such as *EP300* (Yokomizo et al., 2011), *TP53* (Hussain et al., 2007), and *BPTF* (Zhao et al., 2019). Therefore, it’s likely that these nine differential edges present disease-specific change from normal to tumor status.

### Functional Verification of Identified Module Biomarkers

**FIGURE 3:**
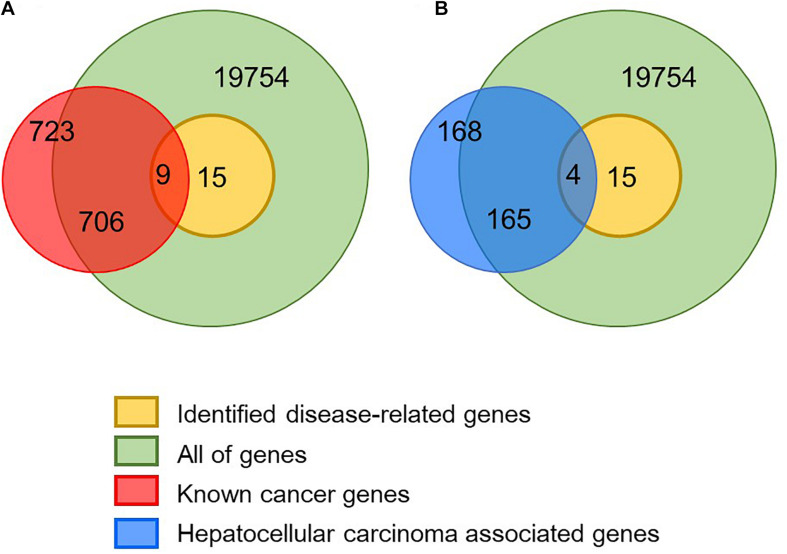
Validation of identified disease module. **(A)** In total, 723 known cancer genes were obtained from the CGC database and nine of them are identified as disease-related genes. **(B)** In total, 168 hepatocellular carcinoma-associated genes were obtained from the KEGG database and four of them are identified as disease-related genes.

In order to verify whether the identified disease-related genes have pathogenic functions, we used known cancer genes for enrichment analysis. In the Cancer Gene Census database, 723 genes have been confirmed to associate with cancer and nine of them are also identified in the module biomarkers (Figure 3 and Table 1). We performed a hypergeometric test and obtained a significant *p*-value of 4.6098×10^−10^, which indicates that the predicted disease-related genes are enriched into the known cancer genes. Meanwhile, we got 531 genes from the cancer pathway in the KEGG database (Kanehisa et al., 2004), four disease-related genes enriched in this pathway, and a *p*-value of 5.5652×10^−4^. In addition, 168 genes of the hepatocellular carcinoma pathway were obtained, and we implemented enrichment analysis with a *p*-value of 6.4041×10^−6^. The results show that the module biomarkers are closely related to HCC.

**TABLE 1:** Common genes between predicted disease-related genes and known cancer genes.

**Cancer-related gene from the CGC database**		**Genes involved in cancer pathways from the KEGG database**	**Genes in Hepatocellular carcinoma pathway from the KEGG database**
*EP300*	*PIK3CA*	*EP300*	*TP53*
*TP53*	*SF3B1*	*TP53*	*CTNNB1*
*POLQ*	*CTNNB1*	*CTNNB1*	*PIK3CA*
*SMARCA4*	*ATM*	*PIK3CA*	*SMARCA4*
*RANBP2*			

**FIGURE 4:**
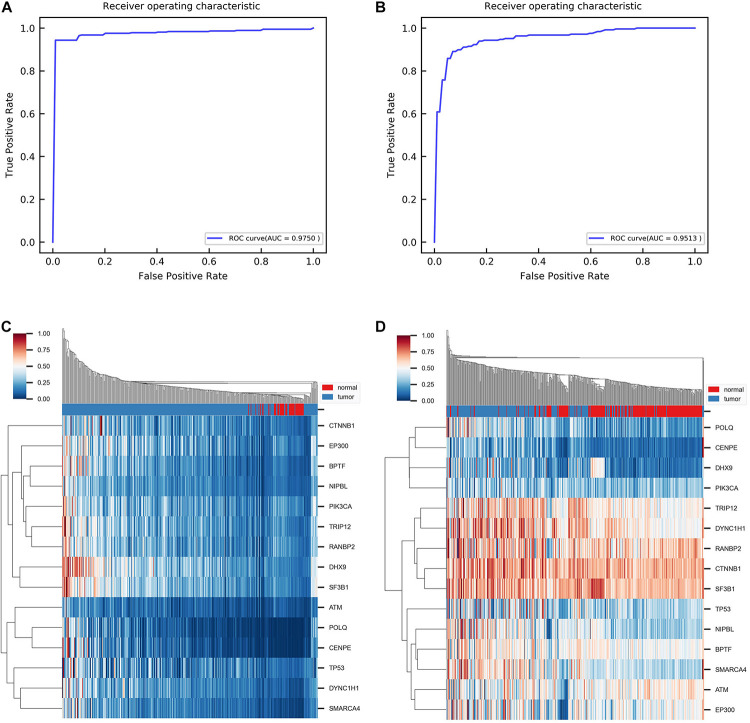
Results of classification and clustering with identified disease-related genes. **(A)** ROC curve obtained from classification between tumor and normal group using fivefold cross-validation. ROC, receiver operating characteristic. AUC, the area under the curve. **(B)** ROC curve obtained from classification between tumor and normal group with independent dataset GSE14520. **(C)** Heat map of hierarchical clustering with single linkage and cityblock distance. **(D)** Heat map of hierarchical clustering with the same parameter in independent dataset GSE14520.

Furthermore, we aimed to test whether the predicted disease-related genes can distinguish the normal samples from the tumor samples. Support vector machine (SVM) algorithms are used to classify samples. To handle the imbalance of samples, we took the random oversampling approach when training the model. Through fivefold cross-validation, the AUC is 0.9750 for the ROC curve (Figure 4A). It indicates that the predicted genes have favorable classification performance. Moreover, we performed hierarchical clustering for all samples with the predicted genes. In the normal sample cluster, 80% of samples were correctly identified (Figure 4C). In addition, we obtained an independent gene expression dataset (GSE14520) for HCC. The same methods were used to validate the predicted genes. Figures 4B,D show that the AUC is 0.9513 for the ROC curve in classification, and 78% of samples of normal sample clusters were correctly identified in hierarchical clustering by the module biomarkers. Besides, when training the SVM model on the original dataset and directly testing it on the independent dataset, the AUC is 0.8735 (Supplementary Figure 5). The above results demonstrate that the predicted disease-related genes can effectively separate tumor and normal samples. It confirmed that the identified module biomarkers are indeed associated with HCC.

## Discussion

In this paper, we put forward an approach to identify the disease-related genes for HCC by constructing a gene regulation network at different levels. In addition, other methods can also identify disease genes. For example, differential expression genes, which were found by measuring the individual differences of gene expression levels, can achieve a better result in sample classification but perform poorly in enrichment analysis. It is not hard to explain that the differential expression genes themselves come from a direct numerical classification between the tumor and normal group; consequently, they make it easier to separate samples into the two groups. However, dramatic changes in expression levels of individual genes may not be the dominant reason for complex diseases, so they cannot be used to identify the cancer genes from the perspective of pathogenic function. Furthermore, some studies are also involved in identifying HCC-related genes. We compared the results of Gui’s (Gui et al., 2015) and Jiang’s (Jiang et al., 2013) methods with our method by the enrichment analysis of the predicted HCC related gene-set. The result of our method is more significant than other methods in CGC database and hepatocellular carcinoma pathway (Supplementary Figure 6). In the CGC database, *p*-values are respectively, 4.6098×10^−10^ (our method), 1.3570×10^−8^ (Jiang’s method), and 0.2577 (Gui’s method). In the hepatocellular carcinoma pathway, *p*-values are 6.4041×10^−6^ (our method), 8.2039×10^−6^ (Jiang’s method), and 1 (Gui’s method). Besides, the three HCC related gene-set were compared in the SVM algorithm on an independent dataset of HCC (GSE39791) (Kim et al., 2014). The AUC of fivefold cross-validation were 0.9131 (Gui’s method), 0.9286 (Jiang’s method), and 0.9663 (our method), indicating the predicted HCC genes of our method can better distinguish normal samples from tumor samples. Gui’s method predicted target genes based on gene expression profile and Jiang’s method identified target genes by PPI network analysis. By contrast, our method also studies the changes in methylation and mutation aspects, which provide more information to identify HCC-related genes.

In this study, we used adjacent non-cancerous tissue samples as normal samples because of few real normal samples in TCGA, and most studies applied the same criteria (Zhao et al., 2018; Ding et al., 2019). In RNA-seq data, 371 tumor samples and 49 adjacent non-cancerous tissue samples were used. The 371 tumor samples were from different individuals, and 49 of them can match adjacent non-cancerous tissue samples.

In the process of network construction, different threshold settings and different *p*-value-adjusted procedures may lead to different results. When we calculated partial correlation to build the networks without indirect edges, we actually calculated the Pearson correlation coefficient and removed non-significant edges at first, then calculated partial correlation for remained edges. In the first step, we aimed to construct basic correlation networks that reflect whether the correlation between any two genes is significant. When the number of tumors and normal samples is unbalanced, the network size for tumor and normal would be seriously skewed, which results in the subsequent analysis being difficult. Consequently, we use a loose *p*-value-adjustment method to reduce the differences of network size between tumor and normal. On the other hand, the selection of candidate genes directly depends on their connected edges number in Diff-PCORN; therefore, it is necessary to minimize the probability of indirect edges. Hence, we used a strict method to adjust the *p*-value of partial correlation to keep a low number of indirect correlations. Besides, some thresholds were used in this manuscript, for example, the disease-related genes were required degree greater than 30 in Diff-PCORN and degree greater than 15 in Diff-MN, Diff-MN was formed by edges whose differential methylation score greater than 0.7. When setting threshold parameter, we used hypergeometric test to ensure thresholds can be selected from a suitable range. When a parameter led to a more significant *p*-value of the hypergeometric test, the parameter was more likely to become the threshold. The results of the hypergeometric test in the CGC databases show changing threshold may affect the number of observed cancer genes, however, the results were all significant (Supplementary Figure 7). In addition, the top 15 genes selected from different thresholds were utilized to classify tumor and normal samples. SVM algorithms with fivefold cross-validation were performed on independent dataset GSE39791. The ROC curve and AUC suggest small changes of thresholds will not bring about a huge difference in classification.

By integrating multi-omics data based on network analysis, we identified a disease module and 15 genes as module biomarkers. All of the 15 genes were highly expressed in the tumor group with *p*-values of *t*-test less than 0.05. Some genes were shown related to HCC, such as *BPTF, DHX9*, and *EP300.* Currently, some genes were few reported for HCC but they were studied in other diseases. For example, DNA Polymerase Theta (*POLQ)* is an error-prone DNA polymerase involved in the replication of damaged DNA and repair of DNA double-strand breaks. In breast tumors, *POLQ* overexpression is considered to favor the emergence and survival of proliferating cancer cells (Lemee et al., 2010). NIPBL cohesin loading factor (*NIPBL*) is the homolog of the sister chromatid cohesion 2 and plays an important role in sister chromatid cohesion, development, DNA repair, and gene regulation. Down-regulation of *NIPBL* impairs the DNA damage response and promotes autophagy. High expression of *NIPBL* is associated with poor prognosis in non-small cell lung cancer (Xu et al., 2015; Zheng et al., 2018). These genes may play a role in HCC due to the similarities in cancer mechanisms.

We applied this method to kidney renal clear cell carcinoma (KIRC) data, using the same thresholds and *p*-value adjusted procedure to build Diff-PCORN and Diff-MN. In Diff-PCORN, 67,355 edges and 19,400 nodes were retained. In Diff-MN, 10,486 edges and 6,857 nodes were kept. Besides, 296 genes were chosen as the first candidate gene set with a degree more than 30 in the Diff-PCORN, 181 genes were selected as the second candidate gene set with a degree more than 10 in Diff-MN, and 47 genes in the overlap between the two candidate gene sets. Furthermore, after ranking 47 overlapped genes, the top 15 genes were predicted as disease-related genes. After performing enrichment analysis with DAVID, the disease-related genes were observed in some pathways, such as Pathways in cancer, Adherens junction, ErbB signaling pathway, and Proteoglycans in cancer (Supplementary Table 7). Besides, 9 genes of our predictions can be markedly observed in the CGC database (*p* = 3.3234×10^−10^), and some genes were related to renal cell carcinoma. For example, *EGFR*, epidermal growth factor receptor, was overexpressed in the majority of clear-cell renal cell carcinoma and co-overexpression of *EGFR* and *erbB-2* gene was associated with metastatic disease (Stumm et al., 1996; Cohen et al., 2007). In addition, *SRC* proto-oncogene was related to the processes of proliferation and survival of cancer cells. The *Src* family was reported to contribute to the appearance of malignant phenotypes in renal cancer cells (Yonezawa et al., 2005; Lue et al., 2015). Although our method was put forward for HCC, the above analysis showed the method of data integration can be applied in other diseases.

There were 108 first-order neighbor nodes with 109 edges for the 14 disease-related genes, and these genes formed isolated modules under the level of first-order neighbors in the normal state (Supplementary Figure 1). The disease-related genes connected 700 nodes with 833 edges and only formed one big module in the tumor state (Supplementary Figure 2). It means that the disease-related genes have more connections and regulations with other genes in the tumor state. The first-order neighbor networks for each disease-related gene are independent and there is no link between any two networks in the normal state (Supplementary Figure 1), but the networks are connected to each other in tumor state (Supplementary Figure 2). From Supplementary Figure 2, each of the disease-related genes can connect some other disease-related genes to constitute a subnetwork. It means these disease-related genes can work together or regulate each other to affect the tumor onset in HCC. In the tumor state, these disease-related genes can regulate some famous oncogenes, like *SETD2* and *STAG1*, but not in the normal state (Supplementary Figure 3). The differential networks show the change of regulations and connections from the normal to tumor state (Supplementary Figures 3, 4). From the differential networks, we can see that the regulations of the identified disease-related genes were changed from normal to tumor, and it is more possible that the disease-related genes take part in the process of tumor development. For example, gene *CREBBP* is a known tumor gene, and it does not show a connection with *PIK3CA* in the normal state (Supplementary Figure 1), but it can be regulated by *PIK3CA* in tumor state (Supplementary Figure 2). This means that gene *PIK3CA* does not directly regulate gene *CREBBP* in the normal state, and *PIK3CA* can affect the tumor gene *CREBBP* in the tumor state.

## Conclusion

In this work, we proposed a method for potential pathogenic gene identification based on networks and multi-omics data integration. By applying our method for HCC, we identified a disease module with 15 potential disease-related genes after integrating data in gene expression, DNA methylation, and SNP levels. The results of classification and clustering demonstrate that the predicted disease-associated genes can distinguish HCC samples from normal samples effectively by both supervised and unsupervised learning. Furthermore, we used known cancer genes from the CGC database and KEGG database to verify the function of the disease-related genes. The significant enrichment results suggest that the predicted disease-related genes can be module biomarkers and are indeed associated with HCC.

## Data Availability Statement

Publicly available datasets were analyzed in this study. This data can be found here: The TCGA-LIHC and TCGA-KIRC dataset for this study can be found in TCGA database (https://www.cancer.gov/tcga). The GSE14520 dataset and GSE39791 dataset for this study can be found in GEO database (https://www.ncbi.nlm.nih.gov/geo/).

## Author Contributions

XL and XC conceived and supervised the study. JX and SS designed experiments. YG and JX performed experiments. SS provided materials and analysis tools. YG and XC analyzed data. YG wrote the manuscript. XL and ZM made manuscript revisions. All authors contributed to the article and approved the submitted version.

## Conflict of Interest

The authors declare that the research was conducted in the absence of any commercial or financial relationships that could be construed as a potential conflict of interest.

## Footnotes

1 https://www.cancer.gov/tcga

2 https://cancer.sanger.ac.uk/census

3 https://string-db.org/

4 https://scipy.org/
