# Supplementary material for: Identification of HCC-Related Genes Based on Differential Partial Correlation Network
Source: Front Genet. 2021 Jul 15;12:672117. doi: 10.3389/fgene.2021.672117 (PMC8320536; doi:10.3389/fgene.2021.672117)
Supplement: Supplementary file 1 [file Data_Sheet_1.docx]

**Supplementary Figure 1.** The subnetwork of PCORN (normal). Disease-related genes and their interaction partners from PCORN (normal) constitute a subnetwork with 109 edges and 122 nodes.

**Supplementary Figure 2.** The subnetwork of PCORN (tumor). Disease-related genes and their interaction partners from PCORN (tumor) compose a subnetwork with 833 edges and 715 nodes.

**Supplementary Figure 3.** The subnetwork of Diff-PCORN. The subnetwork with 790 edges and 677 nodes consists of disease-related genes and their interaction partners from Diff-PCORN.

**Supplementary Figure 4.** The subnetwork of Diff-MN. The subnetwork with 347 edges and 318 nodes is composed of disease-related genes and their interaction partners from Diff-MN.

**Supplementary Figure 5.** Results of classification with identified disease-related genes. ROC curve was obtained from classification between tumor and normal group when training model on original dataset and directly testing it on independent dataset.

**Supplementary Figure 6.** Comparison of results between three methods. **(A)** The result of hypergeometric test. Y-axis is the ratio of significant genes in hepatocellular carcinoma pathway and CGC database. **(B)** Results of classification with HCC-related genes. ROC curve obtained from classification between tumor and normal group using 5-fold cross validation.

**Supplementary Figure 7.** Comparison of results between different thresholds. **(A)** Result of hypergeometric test in CGC database. **(B)** The orange line represents ROC curve obtained from classification with 15 identified disease-related genes. The genes were selected from original thresholds (Diff-PCORN>30, Diff-MN>15, differential methylation score>0.7). Blue and grey line represent the results of classification when threshold of Diff-PCORN changed to 20 and 40, with thresholds of Diff-MN and differential methylation score stay the same. **(C)** The results of classification when threshold of Diff-MN changed to 10 and 20, with other thresholds remained constant. **(D)** The results of classification when threshold of differential methylation score altered to 0.5 and 0.9, with other thresholds remained constant.
