# Supplementary figures and images for: Identification of HCC-Related Genes Based on Differential Partial Correlation Network
Source: Front Genet. 2021 Jul 15;12:672117. doi: 10.3389/fgene.2021.672117 (PMC8320536; doi:10.3389/fgene.2021.672117)

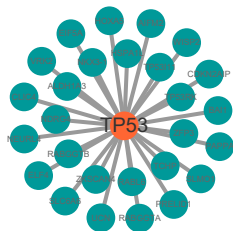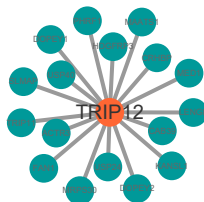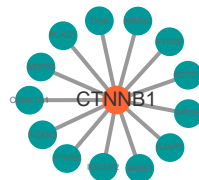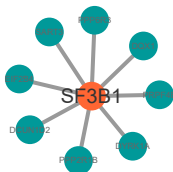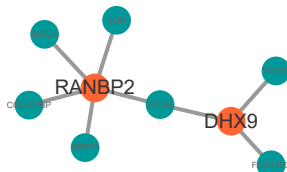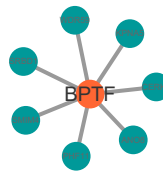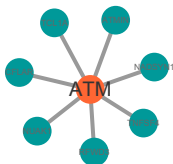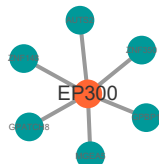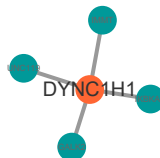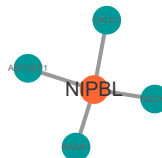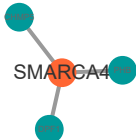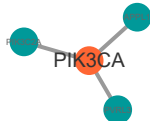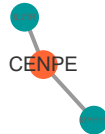

Supplement: Supplementary file 2 [file Image_1.PDF]

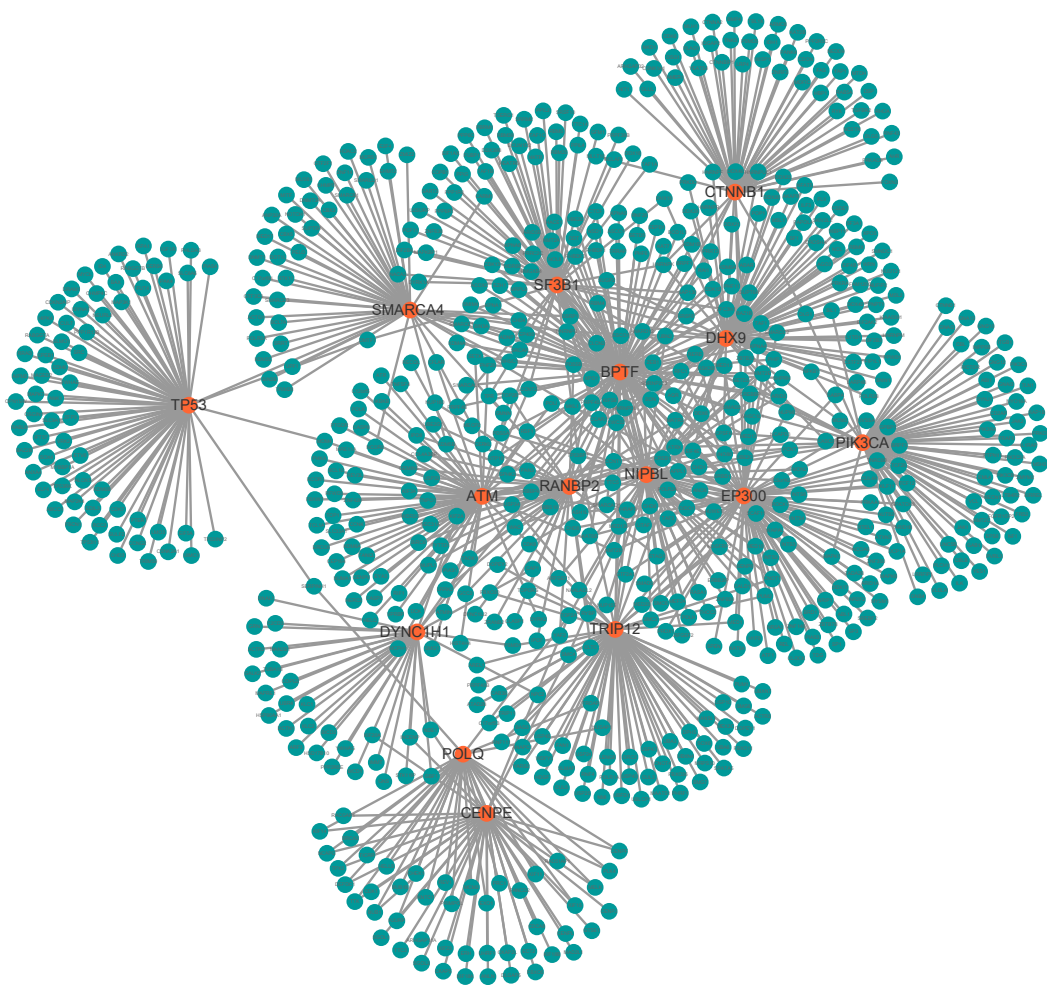

Supplement: Supplementary file 3 [file Image_2.PDF]

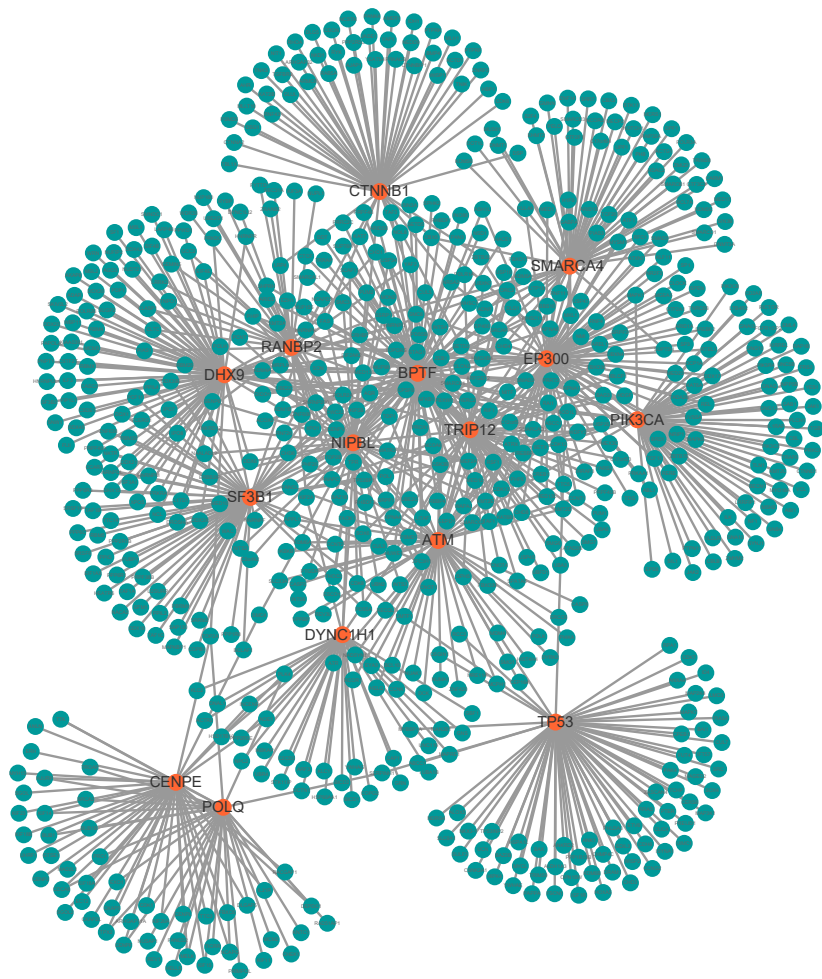

Supplement: Supplementary file 4 [file Image_3.PDF]

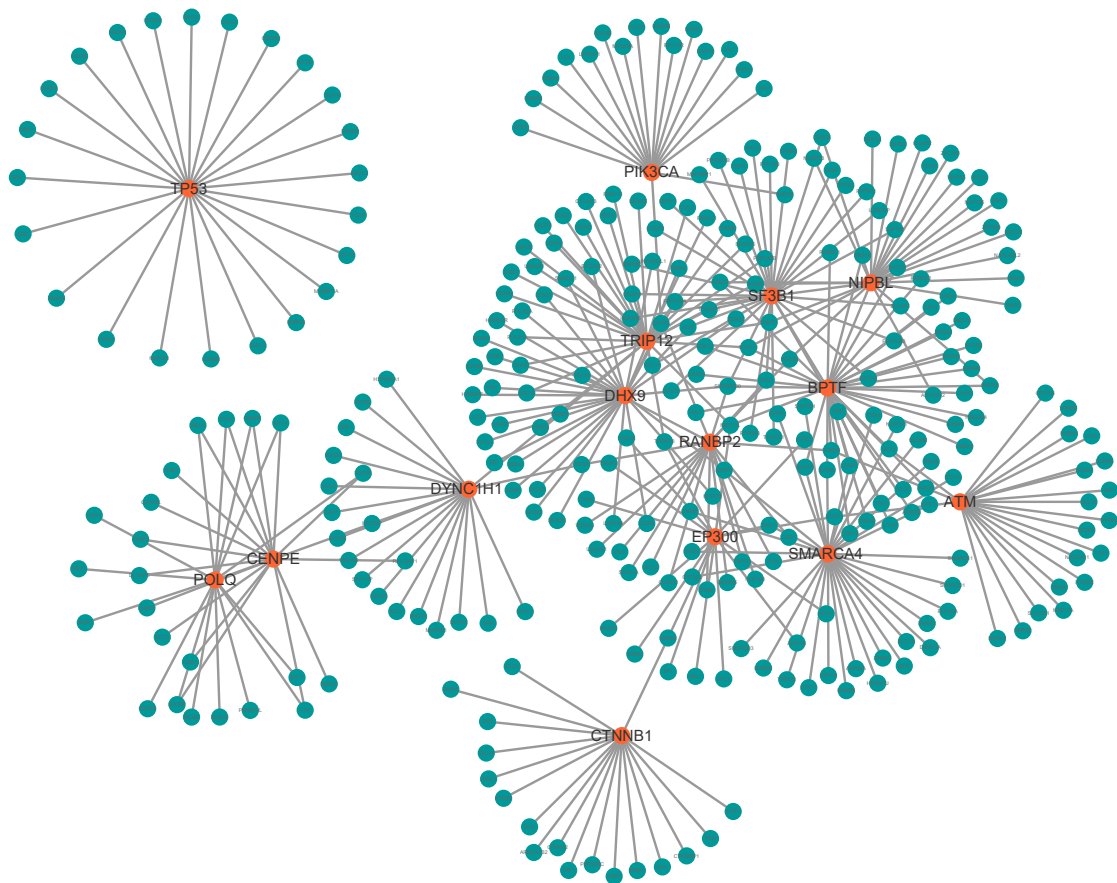

Supplement: Supplementary file 5 [file Image_4.PDF]

Receiver operating characteristic

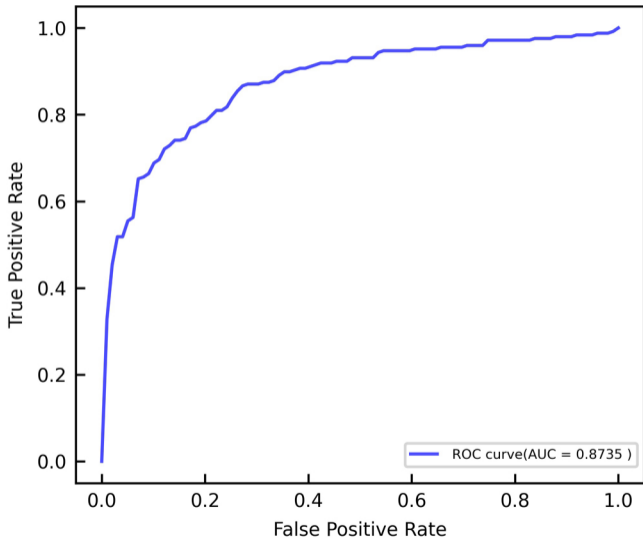

Supplement: Supplementary file 6 [file Image_5.PDF]

A

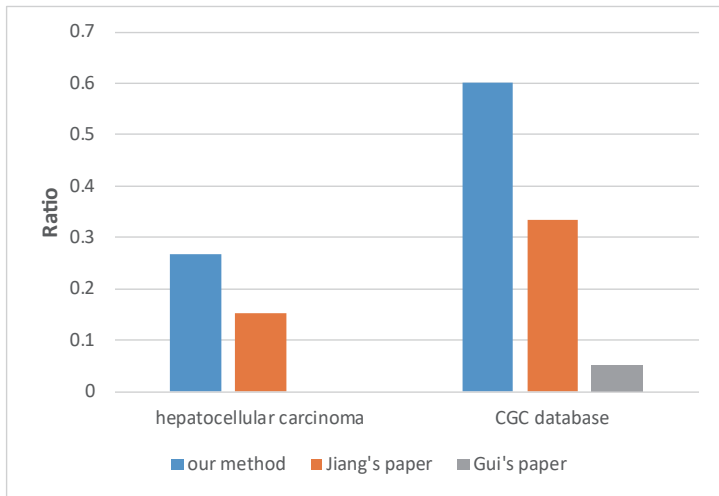

B

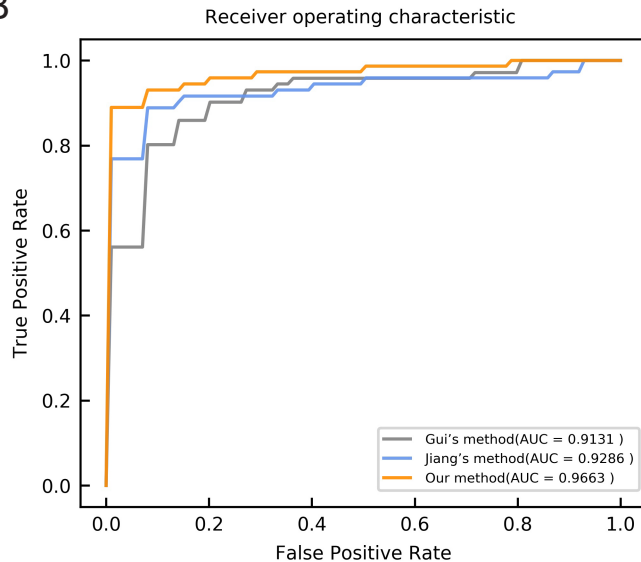

Supplement: Supplementary file 7 [file Image_6.PDF]
