# Supplementary material for: Identification of HCC-Related Genes Based on Differential Partial Correlation Network
Source: Front Genet. 2021 Jul 15;12:672117. doi: 10.3389/fgene.2021.672117 (PMC8320536; doi:10.3389/fgene.2021.672117)

A

Diff-PCORN	Diff-MN	differential methylation score	number of observed genes	*p*-value
20	15	0.7	8	1.5878×10^{-8}
30	15	0.7	9	4.6098×10^{-10}
40	15	0.7	9	4.6098×10^{-10}
30	10	0.7	9	4.6098×10^{-10}
30	15	0.7	9	4.6098×10^{-10}
30	20	0.7	8	1.5878×10^{-8}
30	15	0.5	9	4.6098×10^{-10}
30	15	0.7	9	4.6098×10^{-10}
30	15	0.9	6	8.8786×10^{-6}

B

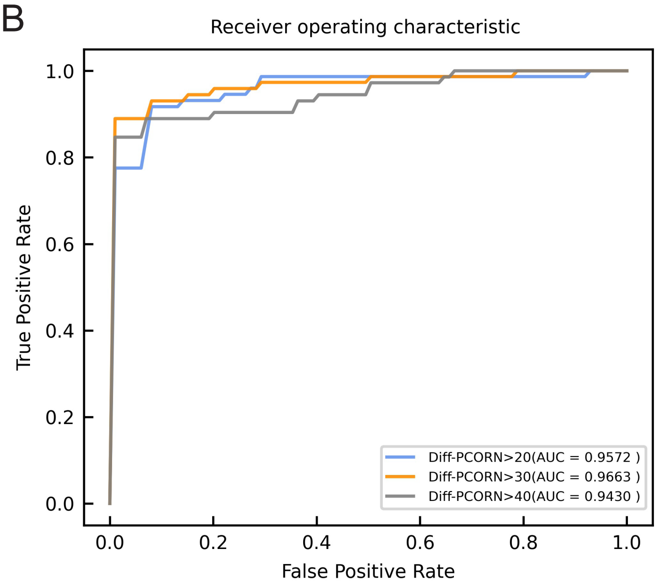

C

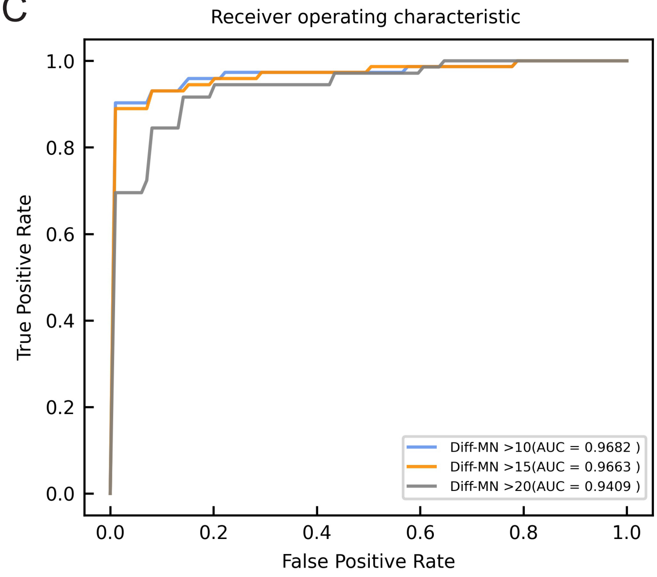

D

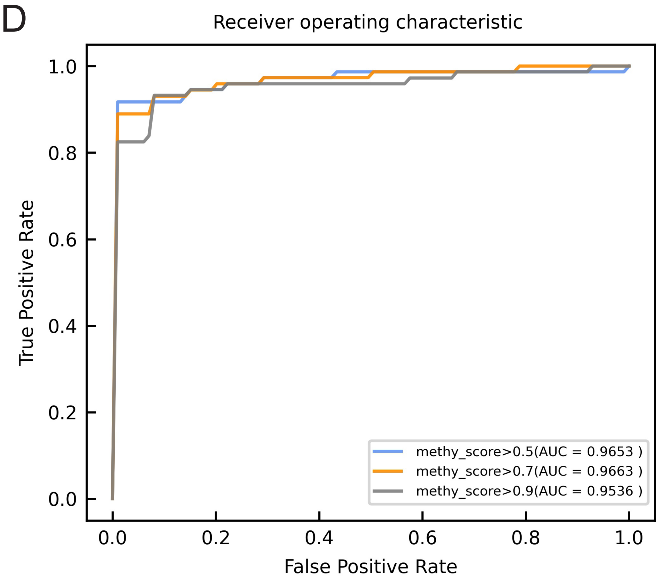

Supplement: Supplementary file 8 [file Image_7.PDF]
